# Smoking Upregulates Angiotensin-Converting Enzyme-2 Receptor: A Potential Adhesion Site for Novel Coronavirus SARS-CoV-2 (Covid-19)

**DOI:** 10.3390/jcm9030841

**Published:** 2020-03-20

**Authors:** Samuel James Brake, Kathryn Barnsley, Wenying Lu, Kielan Darcy McAlinden, Mathew Suji Eapen, Sukhwinder Singh Sohal

**Affiliations:** 1 Respiratory Translational Research Group, Department of Laboratory Medicine, School of Health Sciences, University of Tasmania, Launceston, Tasmania 7248, Australia; sjbrake@utas.edu.au (S.J.B.); Wenying.Lu@utas.edu.au (W.L.); kielan.mcalinden@utas.edu.au (K.D.M.); mathew.eapen@utas.edu.au (M.S.E.); 2 School of Medicine, University of Tasmania, Hobart, Tasmania 7001, Australia; kathryn.barnsley@utas.edu.au

**Keywords:** ACE2 receptor, SARS-CoV-2, Covid-19, Smoking, COPD, Electronic cigarettes, Vaping, Heat-Not-Burn, IQOS

## Abstract

The epicenter of the original outbreak in China has high male smoking rates of around 50%, and early reported death rates have an emphasis on older males, therefore the likelihood of smokers being overrepresented in fatalities is high. In Iran, China, Italy, and South Korea, female smoking rates are much lower than males. Fewer females have contracted the virus. If this analysis is correct, then Indonesia would be expected to begin experiencing high rates of Covid-19 because its male smoking rate is over 60% (Tobacco Atlas). Smokers are vulnerable to respiratory viruses. Smoking can upregulate angiotensin-converting enzyme-2 (ACE2) receptor, the known receptor for both the severe acute respiratory syndrome (SARS)-coronavirus (SARS-CoV) and the human respiratory coronavirus NL638. This could also be true for new electronic smoking devices such as electronic cigarettes and “heat-not-burn” IQOS devices. ACE2 could be a novel adhesion molecule for SARS-CoV-2 causing Covid-19 and a potential therapeutic target for the prevention of fatal microbial infections, and therefore it should be fast tracked and prioritized for research and investigation. Data on smoking status should be collected on all identified cases of Covid-19.

Little attention has been given to the role of smoking in either the transmission of the novel coronavirus, severe acute respiratory syndrome coronavirus 2 (SARS-CoV-2, actual virus) or mortality rate of Covid-19 (name of the disease caused). Smokers contract more respiratory ailments, including colds (commonly rhinoviruses, but also coronaviruses) than non-smokers. Smokers also show double the influenza rate and increased rates of bacterial pneumonia and tuberculosis [1,2,3,4,5]. The damage caused to the lungs by smoking makes patients more susceptible to pulmonary infections, both bacterial and viral [6]. Smokers are 34% more likely than non-smokers to contract the flu [6]. Han and colleagues conclude that literature evidence showed that smoking was consistently associated with a higher risk of hospital admissions after influenza infection [7]. Smoking is the primary etiological factor behind chronic obstructive pulmonary disease (COPD) in the developed world, but environmental pollution and degrading air quality are also responsible in developing countries. It is now the fourth leading cause of death in the world [8]. Vaccination against influenza is strongly recommended for patients with COPD, as the frequency and progression of exacerbations are strongly linked to respiratory viruses in 30% of cases [1]. Rubin et al. found that COPD patients who were prone to viral infections had higher exacerbation rates, more inflammation, and loss of lung function compared to those with existing exacerbating disease conditions [9]. Symptomatology and mortality in influenza-infected smokers were also enhanced [9]. According to the WHO, comorbidities are associated with a high percentage of Covid-19 related deaths [10,11]. In conjunction with the complications arising from comorbidities in patients who smoke [12], we put forth the question of whether smoking, smoking-induced health conditions, and comorbidities, in combination, is culminating in a high risk demographic for both contraction of the virus and the severe presentation of Covid-19.

China has a high male smoking rate at around 50% in rural areas and is estimated to be about 44.8% overall [13]. Most of the deaths identified from the epicenter of the Covid-19 outbreak were in men from older age groups and those with underlying conditions such as chronic respiratory disease, cancer, hypertension, diabetes, or cardiovascular disease. The initial age distribution of Covid-19 cases was skewed towards older age groups with a median age of 45 years (IQR 33–56) for patients who were alive or who had an unknown outcome at the time of reporting. The median age of patients who had died at the time of reporting was 70 years (IQR 65–81) as reported by Sun and colleagues [14]. This data was also supported by an early epidemiological study of 99 Covid-19 cases from Wuhan, China [14]. 

Fatality rates are given as the percentage of the defined group with confirmed Covid-19 that died, and therefore will not add up to 100%. The Table 1 was adapted from Coronavirus Disease (Covid-19) Research and Statistics [15].

The term “coronaviruses” arose from their crown-like appearance when imaged, the Latin for crown being corona. The distinguishing crown-like feature of coronaviruses is attributed to the presence of large type 1 transmembrane spike (S) glycoproteins. This heavily glycosylated cell surface protein contains two distinct functional domains (S1 and S2) which are thought to mediate host cell entry by the virus. The S1 domain contains the angiotensin-converting enzyme-2 (ACE2) receptor-binding domain and is responsible for first stage host cell entry [16]. The S2 domain facilitates fusion between cell and virus membrane, required for cellular infiltration [17]. S proteins are enzymatically modified, exposing the fusion site for cellular adhesion. This is achieved through cleavage by cellular proteases, mediated by protein convertase called “furin” [17,18]. Furin is expressed significantly in the lungs, and respiratory viruses also utilize this system to convert their surface proteins [17]. Although the S protein cleavage site is less observed in coronavirus with similar genomic sequence [17], it is essential to note that more pathogenic influenza viruses share similar cleavage sites [19]. 

The ACE2 receptor provides a human cell-binding site for the S protein for the SARS-coronavirus (SARS-CoV) [20,21,22] (a virus that was first identified in 2003 in a southern province of China [23,24,25]), the coronavirus NL63 [20,26], and now SARS-CoV-2 [27]. Recent studies have found that the modified S protein of SARS-CoV-2 has a significantly higher affinity for ACE2 and is 10- to 20-fold more likely to bind to ACE2 in human cells than the S protein of the previous SARS-CoV [28,29]. This increase in affinity may enable easier person-to-person spread of the virus and thus contribute to a higher estimated R0 for SARS-CoV-2 than the previous SARS virus. The ACE2 protein is expressed on the surface of lung type-2 pneumocytes [30]. It could thus act as a novel adhesion molecule for Covid-19 and be a potential therapeutic target for the prevention of fatal microbial infections in the community. 

An early suggestion is that ACE2 is upregulated on the airway epithelium of smokers. Guoshuai Cai recently reported higher ACE2 gene expression in smoker samples compared to never-smokers. Zhao et al. observed that ACE2 is expressed explicitly in type-2 pneumocytes, in which genes regulating viral reproduction and transmission are highly expressed [31]. This indicates that smokers may be more susceptible to infection by SARS-CoV-2, and possibly Covid-19. We recently identified enhanced ACE2 expression in resected lung tissue from patients with COPD and healthy lung function smokers, albeit comparably less in the latter, while entirely absent in heathy non-smoking individuals (Figure 1). ACE2 expression was quite evident in the type-2 pneumocytes, alveolar macrophages, and the apical end of the small airway epithelium. COPD patients showed significantly higher levels of ACE2, suggesting that COPD further exaggerates ACE2 and potential SARS-CoV-2 adhesion site. ACE2 expression could also be true for patients with another chronic lung disease such as idiopathic pulmonary fibrosis [32]. The attachment of the virus to cell surface ACE2 protects them from immune surveillance mechanisms, leaving them tagged to the host for relatively longer periods, thus making them an efficient carrier and vulnerable host for future infections and spread. The eventual engulfment of ACE2 further provides the virus access to the host cells system, thus providing a flourishing environment, not just to sustain and proliferate but also to mutate and modify host evasion mechanisms. Previous observations using in vivo knockout mice models suggest that SARS-CoV-2 adhesion on ACE2 could also downmodulate the expression of ACE2 itself. This, in turn, increases the production and activation of other related ACE enzymes. This differential modulation and the drastic reduction in ACE2 results in severe acute respiratory failure [33,34].

Wang et al. also noted an ACE2 connection to smoking and Covid-19 [35]. The increases seen in smokers further raises the question of whether this is also true for people engaged in waterpipe smoking [36] and those switching over to the more recent alternatives such as electronic cigarettes and “heat-not-burn” IQOS devices. It is essential to recognize that these devices are not “safer”, they are still a tobacco product that produces vapor or smoke and similarly could cause infectious lung damage as we see with traditional cigarettes [37,38,39]. 

Further research on these products and their influence on the virulence of coronaviruses is urgently needed. Following the outbreak in New York City, Mayor Bill de Blasio announced that “If you are a smoker or a vaper that does make you more vulnerable,” urging that now is the perfect time to quit [40]. Smokers, as a vulnerable group, must be supported to quit and should be advised to avoid areas where they may be liable to be exposed to Covid-19, especially smokers with pre-existing respiratory health concerns. Smokers should be prioritized for vaccination when a vaccine is developed, particularly if it is found they are a key transmission source. 

Research on smoking and potential exacerbations of Covid-19 transmission and mortality should include waterpipes, electronic smoking devices, and “heat-not-burn” devices, such as IQOS devices. Further compounding this link between smoking and Covid-19 vulnerability are the comorbidities that have been identified as a significant increased risk factor for severe and fatal Covid-19. The link between smoking and comorbidities, such as diabetes and cardiovascular disease, have long been established [12]. As a research community, we must ask the questions:

(1) Are COPD and other smoking-related illnesses associated with fatal Covid-19 cases?
(2) Are smokers more likely to contract and transmit SARS-CoV-2 than non-smokers?
(3) Are demographics with high smoking rates more vulnerable to Covid-19 outbreaks?

WHO and all countries should ensure that the smoking status of patients identified with Covid-19, including deaths, is recorded and incorporated in data sets, so the smoker’s relationship to Covid-19 can be determined. 

Status data collection could be simple in four categories,

1. active smoker,
2. passive smoker (those living in households with smokers or working in smoky environments),
3. former smoker (12 months or longer abstinence),
4. non-smoker.

Governments should act to reduce smoking rates in all countries in accordance with the WHO Framework Convention on Tobacco Control (FCTC), and initiate a stimulus package for health, as they have done for business, at the time of this outbreak/pandemic including all communicable pulmonary diseases and Covid-19, as it is possible that smoking exacerbates contraction, transmission, and mortality. It appears that smoking has the potential to upregulate the ACE2 receptor, making smokers and COPD patients more vulnerable to Covid-19. The new electronic smoking devices also do not seem to be safer options. ACE2 thus could be a potential therapeutic target for SARS-CoV-2 and should be prioritized for further research.

## Figures and Tables

**Figure 1:**
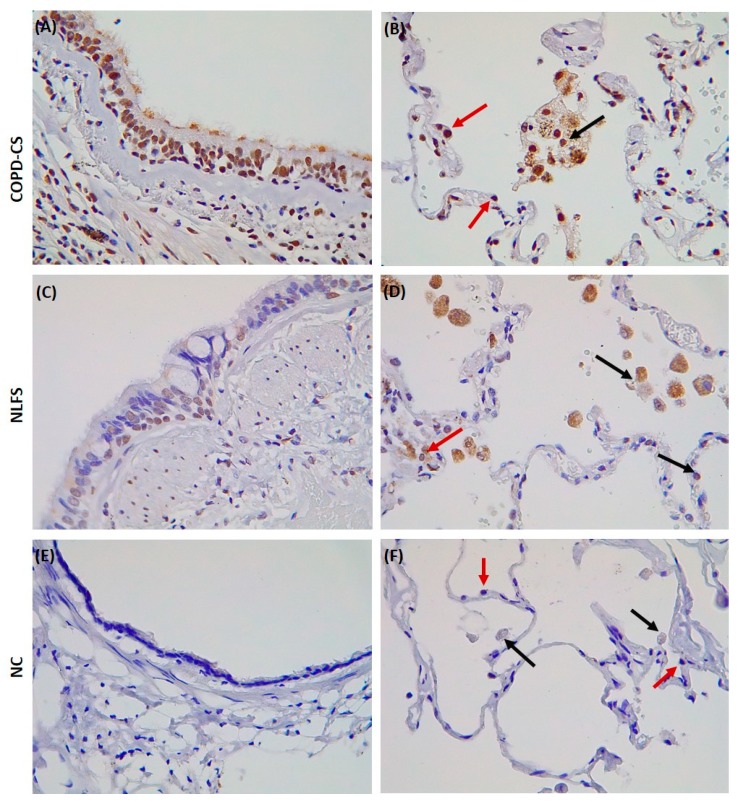
Surgically resected lung tissue stained for the angiotensin-converting enzyme-2 (ACE2) receptor. Current smoker with chronic obstructive pulmonary disease (COPD-CS), (**A**) showing positive staining in the small airway epithelium but also apical including cilia (**B**) red arrows indicating positive staining in type-2 pneumocytes and black arrows showing alveolar macrophages positive for the ACE2 receptor. Normal lung function smoker (NLFS), (**C**) and (**D**) showing similar pattern for COPD-CS although a little less staining is observed. Normal controls (NC), (**E**) and (**F**) no staining observed in any of the areas. **This is the first immunohistochemical human lung evidence for ACE2 receptor expression in smokers and patients with COPD.**

**Table 1:** Risk factor-based fatality rates of Covid-19 from early data in China.

**Age group**	**Fatality rates**
0–9 years	0%
10–19 years	0.2%
20–29 years	0.2%
30–39 years	0.2%
40 - 49 years	0.4%
50–59 years	1.3%
60–69 years	3.6%
70–79 years	8%
80 years and above	14.8%
**Underlying health conditions**	
Cardiovascular disease	10.5%
Diabetes	7.3%
Chronic respiratory disease	6.3%
Hypertension	6%
Cancer	5.6%
No underlying health conditions	0.9%
